# A Novel Method for Understanding the Mixing Mechanisms to Enable Sustainable Manufacturing of Bioinspired Silica

**DOI:** 10.1021/acsengineeringau.2c00028

**Published:** 2022-11-16

**Authors:** Yahaya D. Baba, Mauro Chiacchia, Siddharth V. Patwardhan

**Affiliations:** Green Nanomaterials Research Group, Department of Chemical and Biological Engineering, University of Sheffield, Mappin Street, SheffieldS1 3JD, U.K.

**Keywords:** nanomaterials, scale-up, mixing time, degree of mixing, mixing kinetics

## Abstract

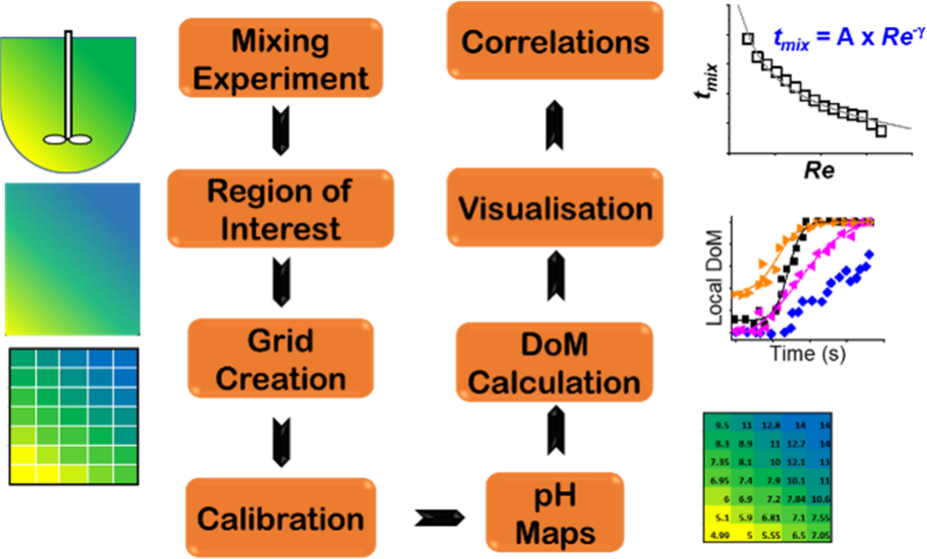

Bioinspired silica (BIS) has received unmatched attention in recent times owing to its green synthesis, which offers a scalable, sustainable, and economical method to produce high-value silica for a wide range of applications, including catalysis, environmental remediation, biomedical, and energy storage. To scale-up BIS synthesis, it is critically important to understand how mixing affects the reaction at different scales. In particular, successful scale-up can be achieved if mixing time is measured, modeled, and kept constant across different production scales. To this end, a new image analysis technique was developed using pH, as one of the key parameters, to monitor the reaction and the mixing. Specifically, the technique involved image analysis of color (pH) change using a custom-written algorithm to produce a detailed pH map. The degree of mixing and mixing time were determined from this analysis for different impeller speeds and feed injection locations. Cross validation of the mean pH of selected frames with measurements using a pH calibration demonstrated the reliability of the image processing technique. The results suggest that the bioinspired silica formation is controlled by meso- and, to a lesser extent, micromixing. Based on the new data from this investigation, a mixing time correlation is developed as a function of Reynolds number—the first of a kind for green nanomaterials. Further, we correlated the effects of mixing conditions on the reaction and the product. These results provide valuable insights into the scale-up to enable sustainable manufacturing of BIS and other nanomaterials.

## Introduction

There is an explosion in nanomaterials discovery and synthesis, leading to many current and potential applications.^1^ Their unique properties (e.g., high surface area, catalytic reactivity, optical response, etc.) offer high performance desired in many applications, e.g., catalysis, adsorption, electronics, energy harvesting/storage, etc.^2,3^ While many new nanomaterials are being discovered, very few have been manufactured at scale, and hence, their vast potential is not realized.^4−6^ The reasons for such a staggering lack of impact have been attributed to the lack of scalability and unfavorable economics.^4,7^ Mainly due to the conditions or reagents used, scale-up is not feasible or causes significant loss in performance. Further, most methods for high-value nanomaterials processing are energy and resource intensive, which in turn means that they are expensive and wasteful.^8^ These limitations have created an urgent need for sustainable, controllable, and scalable methods for nanomaterials.^9,10^

To this end, the synthesis of silica inspired by biosilicification, which employs green chemistry principles, has witnessed unprecedented attention, expansion, and application in recent years.^7,11^ This route aims to produce silica with controlled and well-defined properties and is carried out under mild conditions as compared to conventional methods (e.g., sol–gel processing, colloidal synthesis, hydrothermal synthesis, and polyol methods). The bioinspired silica (BIS) synthesis uses a bioinspired additive (typically an organic amine) and operates at neutral pH, ambient temperature, and in aqueous solution with short reaction times of 5–15 min.^12^ The importance of BIS synthesis lies in its potential for sustainable, economical, and scalable manufacture of highly desirable mesoporous and other grades of silica with a wide range of applications.^13−16^

To date, BIS synthesis has been successful mostly at small scales. While its potential to scale-up has been identified,^13^ large-scale manufacturing has not been achieved. This is because BIS synthesis follows nonclassical (and yet not fully understood) formation pathways, which includes hydrolysis, reactive self-assembly, polymerization, and condensation, leading to oligomerization/nucleation of primary particles (typically 5–10 nm), followed by aggregation (to secondary particles, typically 200–400 nm) and agglomeration (to form tertiary particles >1 μm).^17^ The speciation of each step is not yet identified and their timescales are still unknown. As such, the effects of production scales on the process chemistry have not been clearly understood. Specifically, the multistep reactions in silica formation, each with distinct rates, are influenced by mixing, and in turn, by production scales, leading to variation in BIS properties with scale-up.^18^ It is therefore important to understand the mixing mechanisms underpinning BIS synthesis to control final product characteristics and to develop predictable strategies for upscaling the process from the laboratory scale to an industrial scale.

Indeed, mixing operation is known to play a vital role in a wide range of industrial applications, and this ranges from chemical and pharmaceutical to food industries as it plays a major role in controlling the product quality in many reactions, including precipitation,^19−21^ organic synthesis,^22−24^ and polymerization reactions.^25,26^ A key parameter necessary for scale-up and design of suitable reactors is the mixing time.^27^ In particular, successful scale-up can be achieved if mixing time is measured, modeled, and kept constant across different production scales. The importance of mixing in homogeneous reactions/processes was first introduced by Danckwerts^28^ and later demonstrated experimentally.^29^ The associated mixing theory is briefly summarized below before discussing its application in nanomaterials synthesis.

In a stirred tank system, the most important fundamental quantities are power number (*P*_O_), power draw (*P*), and the impeller Reynolds number (*Re*) represented by the following equations
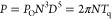
1
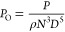
2
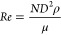
3

There are three mixing mechanisms: macromixing, mesomixing, and micromixing.^21^ Macromixing which is the blend time in a system caused by mechanical stirring is defined as the mixing on the largest possible scale.^30^ Micromixing is mixing on the smallest scales of motion (the Kolmogorov scale) dominated by diffusion. Mesomixing, on the other hand, is the turbulent dispersion of a feed stream shortly after it enters a mixing vessel caused by the action of the bulk fluid interacting with the feed stream.^31^ To develop a scale-up strategy, it is important to identify which mixing mechanism is controlling the system.

There is a general agreement in the published work that the mixing time in stirred tank reactors (STRs) is proportional to the agitator speed, *N*, the power consumed, *P* per unit liquid volume *V* (and hence, energy dissipated, ε) and, in turn, to Reynolds number, *Re*,^27,32−35^ as follows
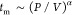
4
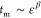
5
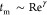
6

Constants α and β are typically −0.33 under turbulent mixing (or mesomixing involving large eddies) for a wide range of impellers.^33,34,36^ β is about −0.5 under micromixing conditions in tank reactors (engulfment) or in micro- and milli-reactors.^33,35,37^ However, α and β have been reported to vary in the range of −0.167 to −0.54 depending on the dominant mixing mechanisms.^33^ For example, Ghotli et al. found that β was around −0.2 for a range of agitator designs.^38^ Such variations can be attributed to measurements of and the definition used for the degree of mixing (DoM) as well as the conditions and geometries investigated.^33^ When considering eq 6, it has been reported that in the laminar and transient regime, mixing time decreases with *Re* (γ is negative), while it remains somewhat independent of *Re* in the turbulent region.^34,39^ We will return to this point in the Results section.

### Effect of Mixing in Nanomaterials Synthesis

The complexities of interactions between mixing and fast precipitation or crystallization reactions as noted by Marcant et al.^19^ have been investigated for various chemical systems, such as inorganic crystallization processes, precipitation process that involves instantaneous irreversible reaction of ionic solutions, and their subsequent crystallization, and nanoparticle synthesis in microfluidic reactors.^40−43^ Despite the proven critical role that mixing and fluid dynamics play in product quality, the identification of mixing mechanisms and quantitative analysis of mixing in such systems have received little attention in the literature. Selected examples from the literature are highlighted below.

Gutierrez et al.^44^ investigated the effect of mixing performance on the production of nanomaterials. They carried out the synthesis of silica nanoparticles by comparing the product in micromixer–microreactor and batch reactor systems. The authors pointed out that in Stöber synthesis of silica nanoparticles, the microreactor produced much narrower particle size distributions with higher reactant conversion than batch reactors. The silica synthesis in agitated vessels has also been reported.^45−48^ For example, Tourbin et al. and Schaer et al.^46,47^ developed a method for the destabilization of a silica sol in a batch agitated vessel, and Baldyga et al.^45^ experimentally investigated the batch process for precipitated silica. While these studies focused on the effects of mixing on the silica *aggregation* process, there are no previous reports on quantifying mixing mechanisms for silica *synthesis*. In particular, investigation of the effects of mixing on the reaction and, in turn, the properties of silica produced are missing.

Beyond silica, other nanomaterials were also investigated. For example, Kim and Tarbell^49^ experimentally and theoretically studied the effects of turbulent mixing on barium sulfate precipitation in a semibatch reactor. Their findings showed that precipitation was affected by the impeller rotational speed dominated by mass transfer to particle rather than micromixing of the feed streams. Kisyelova et al.^50^ studied the effects of reactor configurations on the production of silver nanoparticles. Their work showed that the intensification of the mixing process and the use of a spinning disc reactor results in a decrease of the nanoparticles’ average size.

It is important to note that material syntheses, silica synthesis process in particular, are often complex, involving a series of reactions that span across timescales overlapping with mixing timescales. This implies mesomixing or micro- and mesomixing as the underpinning mechanism. However, while some progress has been reported for mesomixing theory and its application, it remains limited to nonreactive systems (e.g., antisolvent precipitation) or reactions that are much faster than mixing (e.g., BaSO_4_ formation). Therefore, there is a significant need to apply mixing theory to specific cases, such as the green synthesis of nanomaterials to enable their scale-up.

### Techniques to Quantify Mixing

As noted above, quantitative understanding of underpinning mixing mechanisms is limited for complex chemistries with slow/multistep reactions that are typically required for the synthesis of high-value nanomaterials. However, for traditional reactions, research in recent years has been carried out to quantify the mixing time and mixing efficiency using a range of measurement methods. These generally fall under intrusive and nonintrusive techniques that are comprehensively discussed elsewhere.^51−54^ Of many experimental techniques used to investigate mixing times in stirred tank reactors, colorimetric methods are easy to use, rapid, nonintrusive in nature, and versatile in application to a wide range of reactor geometries.^51^ In such studies, acid–base neutralization reactions with indicators, such as phenolphthalein and bromothymol blue, are popular.^53,55−60^ With significant improvements in image acquisition and automated analysis, colorimetric methods using high-resolution image analysis are emerging as promising methods to study mixing as recently demonstrated.^56,61^ Further, these techniques can additionally provide dynamic and localized information of mixing, yet offering accuracy that is comparable with conductivity measurements as a benchmark.^62^ However, the use of colorimetric methods with high-resolution image analysis has not been utilized yet for studying mixing dynamics and mixing mechanisms. For example, one study^56^ focused only on one feed location and hence could not map the effects of fluidic parameters, which in turn precluded any quantitative analysis of the mixing mechanism. Further, it is not clear if this method is suitable for complex multiphase reacting systems such as BIS formation.

To address these points, herein, we report the development of a hybrid colorimetric technique for probing the effects of mixing on the formation of bioinspired silica (BIS). The technique is based on the principle of video recording from a high-speed camera and image processing linked with pH changes upon a fast acid–base reaction in transparent vessels using scripting software, such as MATLAB for image analysis. This technique is capable of providing information on local and global degrees of mixing within the vessel. Upon validation, the technique was then used to probe mixing mechanisms in BIS formation by investigating the effects of stirring speed and feed location to identify the dominant mixing mechanism. The degree of mixing and BIS properties were measured, and their dependence on the reaction and the process conditions were mapped to develop a quantitative relationship suitable for scale-up in the future. In particular, we aim to:

(1) Identify dominant mixing mechanisms controlling BIS synthesis.
(2) Develop an in-house method for image acquisition and analysis to calculate the global and local mixing times and overall degree of mixing or homogeneity (DoM).
(3) Study the mixing kinetics for BIS formation reactions over a wide range of mixing scenarios.
(4) Explore the correlation between the mixing time and fluidic conditions and compare them with established theory.

## Experimental Methods

### Materials, Synthesis, and Preparation

The bioinspired silica synthesis is dependent on the degree of dissolution and mixing of two or more starting materials: a silica precursor (sodium silicate in this case) and a bioinspired additive (tetraethylenepentamine (TEPA)). The synthesis of bioinspired silica was achieved by following a procedure described in the literature.^12^ Experiments were conducted using a 1 L Radleys Reactor-Ready setup made up of a cylindrical vessel. The ratios between the vessel diameter *T*, impeller diameter *D*, tank height *H*, and the impeller clearance *C* are as follows
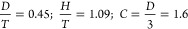
7

The synthesis involved injecting 1 M HCl into a premixed aqueous solution containing TEPA and sodium silicate (30 mM, Si/N molar ratio of 1). A bromothymol blue indicator, used to monitor reaction progress and spatial mixing, was also added to the premixed solution (20 mg/L). The addition of acid (within 5 s) triggers the reaction via neutralization of a high-pH premix aqueous solution (from ≈pH 13 down to pH 7 ± 0.05). This acid addition is considered very fast compared to several minutes required for completion of the reactions at small scales. An unbaffled 1 L classic stirred tank reactor with a 45° pitched blade impeller was used to carry out the reaction at room temperature for 5 min. An unbaffled reactor was preferred as a model system to focus on understanding mixing effects instead of optimizing mixing. After 5 min of reaction, the reactor was drained into sample tubes and the samples were centrifuged at 5000 rpm for 10 min. The supernatant was stored separately for chemical analysis, and the precipitate was resuspended in deionized water. Another two rounds of centrifugation and washing were undertaken before drying the samples at 80 °C overnight.

**Figure 1:**
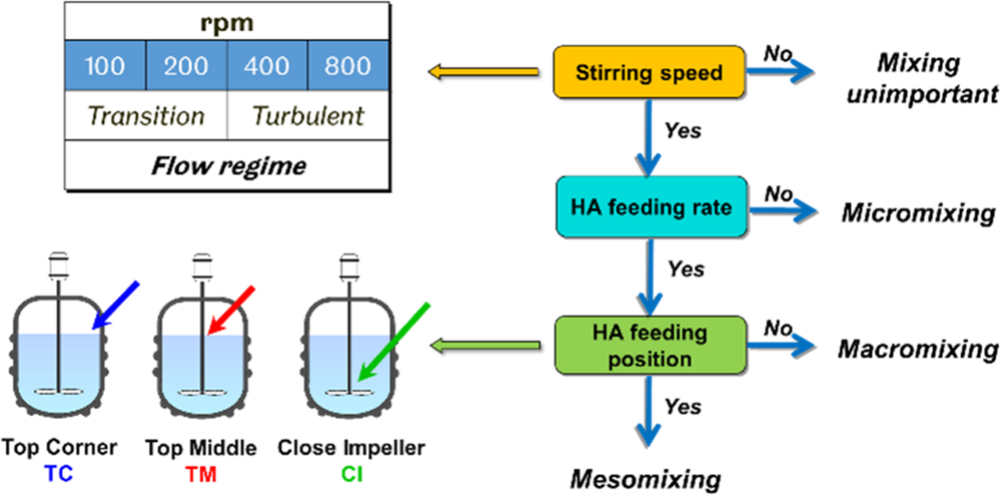
Flowchart of the procedure and range of each individual operating parameter for mixing characterization.

An experimental procedure adapted from the seminal work by Bourne^63^ was used to identify the mixing mechanism underpinning BIS synthesis (Figure 1). This protocol is based on the use of Damkohler number (*Da*), which compares mixing and reaction timescales and can help identify a dominant process. For complex reactions, such as BIS synthesis, where the measurement of kinetics is impractical, this protocol allows the identification of the rate-limiting process without the need for explicitly measuring *Da*. Another advantage of this methodology is that it helps identify the key mixing mechanism that is agnostic to growth mechanisms, and hence, it becomes highly relevant to reactions following nonclassical formation pathways, such as BIS synthesis.

Specifically, the procedure includes a range of stirring speeds, feed (1 M HCl in this case) rates, and feed locations. This procedure simultaneously enables the identification of the dominant mixing mechanism as well as maps a range of fluidic conditions to develop mixing correlations. Our earlier work has shown that varying the rate of acid addition has an effect on the reaction and product characteristics;^12^ hence, this study focused on the effects of stirring speed and acid inlet position.

### Characterization

To study the effects of mixing on silica, we measured the porosity, silicon speciation (monomers, oligomers, and polymers), and morphology of silica produced under different conditions as described previously.^12,64^ A Micrometrics porosimeter was used to perform nitrogen adsorption and the isotherms produced were used to calculate the Brunauer–Emmett–Teller (BET) specific surface area, pore volume, and BJH pore sizes. A silicomolybdate colorimetric assay was used to quantify the conversion of silica precursor as well as the formation of silica. The morphology and particle size distribution of silica were examined by transmission electron microscopy (TEM).

### Image Acquisition and Processing

**Figure 2:**
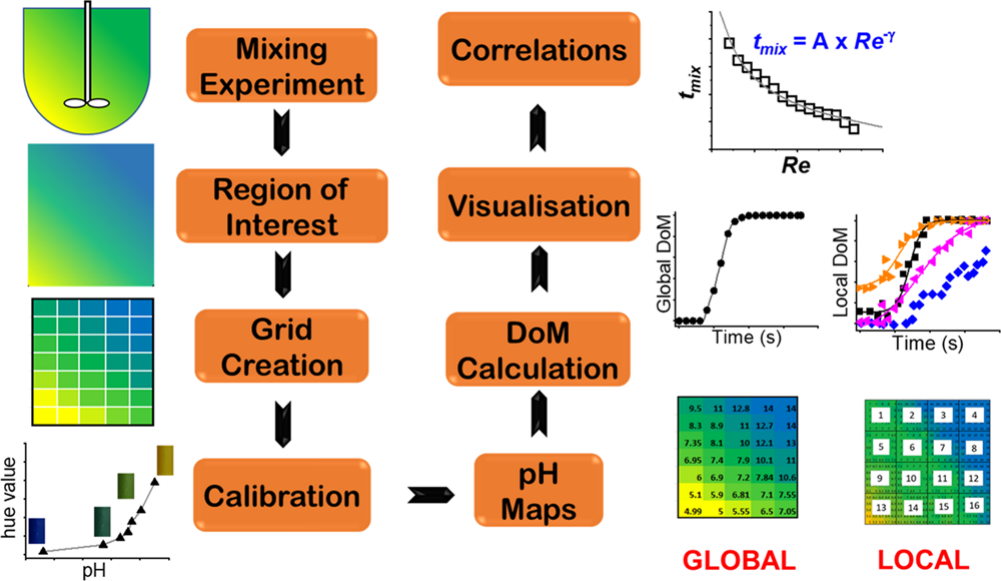
Strategy for quantifying the degree of mixing (DoM) for global and local mixing and obtaining mixing correlations.

A diagrammatic representation of the strategy adopted for image analysis, degree of mixing quantification, and probing mixing mechanism is presented in Figure 2 and described below.

#### Image Acquisition

The reaction was monitored using a high-speed Baumer 1.5 MP camera. For each experimental condition, videos were recorded spanning the duration of the reaction and were saved as a sequence of image frames in TIFF format with a resolution of 1920 × 1080 pixels using a digital color camera. A white vinyl sheet was placed behind the mixing vessel to ensure a consistent image background to allow for easy color identification. Care was taken in image acquisition during experiments to ensure uniform diffuse lighting (Fovitec StudioPRO—Daylight 600 LED panel) with minimal glare, and image preprocessing was not necessary.

#### Generation of pH Map and Instantaneous Degree of Mixing

Generating a homogeneity or pH map for each image is essential in determining the instantaneous degree of mixing (DoM) in the vessel for the duration of the experiment. Following the literature, DoM was defined as the mixing time necessary to achieve 95% homogeneity starting from an initially inhomogeneous mixture.^55,65,66^ This is the same as the time needed to reach 5% from perfect mixing as defined by others.^27,30,55,67^ The images were processed using the RGB color model using a code written in MATLAB R2019b (Mathworks). In the RGB color model, a color can be represented as a combination of varying hue levels ranging from 0 to 255 of pure red, green, and blue light. The area of interest was selected and represented in MATLAB by a matrix of pixels *P*(*i*,*j*,*t*), which were separated into three RGB components or channels i.e.,: *R*(*i*,*j*,*t*), *G*(*i*,*j*,*t*), and *B*(*i*,*j*,*t*). The images were read in sequence, each cropped to a suitable region of interest (ROI), and subsequently subdivided into a grid of (*m* × *n*) subimages with each grid having (*i* × *j*) pixels. Therefore, the total number of pixels per grid is related to the size of the image as follows: *V* = *m* × *i* and *H* = *n* × *j* with *V* and *H* being the number of vertical and horizontal pixels that make up each image frame and *V* × *H* is the resolution of the image frame in pixels. Only one RGB component can be used to analyze the region of interest, and the decision on which was used here is presented in the next section.

To determine the dominant color produced by the indicator in any grid (between blue to yellow representing a pH range of ∼13.00–5.0), a histogram of the pixel values in that grid was produced, and depending on the location of the peak, a pH value was assigned to that grid using a pH color calibration (see the Results section). Using the calibration, a pH map of size (*m* × *n*) can be constructed for every image frame from which a characteristic average pH can be calculated for each frame as follows
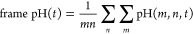
8

As care was taken when setting up the experiments to minimize glare, only around 3% of the typical ROI contained glare. For grids with a glare, the grid was assigned a value that corresponds to that of the neighboring grid immediately preceding it. We can therefore calculate the degree of mixing (DoM) of the mixture at any given time directly from the average frame pH
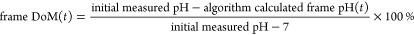
9

It is worth noting that the frame pH and frame DoM are global variables that describe the mixing condition at a particular time instant and give no information on spatial mixing patterns and local dead zone spots, where there is little or no mixing. To obtain such information, the averaging scheme was modified so that the mean pH is calculated not for the whole frame but for (*p* × *q*) segments in each frame or ROI. Therefore, eq 10 used for segment pH now becomes
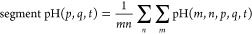
10

### Parametric Study

To determine the mixing time, the time-dependent evolution of degree of mixing was analyzed using the Boltzmann sigmoid equation in OriginPro (see eq 11). This method has been previously validated to study the hydrodynamics of a mechanically stirred anaerobic sequencing batch biofilm reactor (ASBBR).^68^
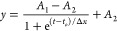
11where *y* is the degree of mixing (DoM).

*A*_1_ is the initial *y* value (*y*(−∞)),

*A*_2_ is the final y value (y(+∞)),

*t*_*o*_ is the degree of mixing value in % corresponding to halfway between *A*_1_ and A_2_.

(*y*(*t*_*o*_) = (*A*_1_ + *A*_2_)/2).

Δ*x* is the size of the value span in time that has the largest variation in *y* (DoM).

The data was fitted to the Boltzmann sigmoidal model using the Lenvenberg–Marquardt iteration method. The fit parameters were noted for comparison between systems/experiments and further analyzed. The mixing time, *t*_m_, was defined as a time required to reach 95% perfect mixing, and it was calculated by solving eq 11 using the fit parameters.

## Results and Discussion

### Effect of Mixing on BIS Formation and Properties

To identify the key mixing mechanisms for BIS synthesis, we utilized the workflow shown in Figure 1. For the stirring speeds and feed locations shown in Figure 1, we performed BIS synthesis. The effects of mixing (impeller stirring speeds and the location of feed addition) on the formation and properties of BIS were studied. In particular, we monitored the speciation as a function of mixing conditions by measuring the solution concentrations of silicate monomer, oligomers, and polymers (i.e., the silica formed) at the end of every reaction. This can provide insights into mixing effects on the BIS formation pathways. We also measured key quality attributes for silica—particle size (and size distribution), porosity (surface area, pore sizes, and pore volumes) and yield.

**Figure 3:**
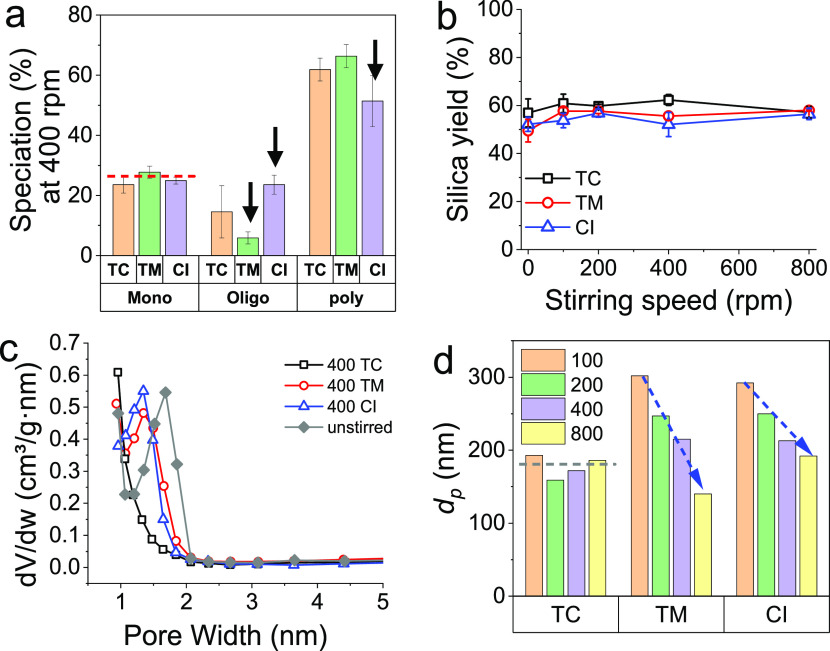
Effect of stirring speed and of feeding position on (a) silicate speciation (monomer, oligomers, and particles), (b) silica yield, (c) pore size distribution, and (d) particle sizes (obtained from Figure 4).

The speciation of silicates—distribution of monomer (which is the precursor), oligomers (the intermediates) and polymers (silica particles)—is shown in Figure 3a for a representative case of 400 rpm impeller rotation speed (see Figure S1 for the entire data set). The amount of monomer was found to stay within the experimental error (see red dashed line in Figure 3a), which suggests that the first reaction, the conversion of the monomer to oligomers, was not affected by the stirring speeds or feeding positions investigated. Interestingly, there is a feed-point effect on the oligomer population for the top-middle and close-to-impeller positions. While the population of oligomers reduced for the top-middle (TM) position, it increased for the close-to-impeller (CI) position. Commensurate changes to polymer populations were seen in both cases. This suggests that the degree of homogenization of acid is affecting the balance between the oligomer formation (from monomers or from particle breakage) and consumption reactions, which is consistent with the literature.^69^ Although the effect on speciation was modest, the timescales for the reaction involving oligomers could be of the same order as the timescales of mixing. Surprisingly, when the solids were collected after centrifugation, dried, and analyzed for yield, it was found that neither the impeller speed nor the feed location affected the yields beyond experimental errors (Figure 3b). Future research is focusing on detailed measurements of the chemical kinetics of these reactions, which can then be combined with mixing models.

Next, the porosity of the samples was probed using gas adsorption. Note that for silica, porosity is one of the most important characteristics in many applications, such as adsorption, separations, and fillers. We measured the BET specific surface area and BJH pore sizes. While there were no significant changes to the surface area or pore volumes as a function of impeller speed or feed location (data not shown), the pore sizes varied with the mixing conditions. The results in Figure 3c on pore size distribution show that most of the samples were micro- and mesoporous (pore widths under 50 nm). The pore sizes changed slightly between unstirred (∼2 nm) and stirred conditions with feed location of CI and TM (∼1.5 nm). However, when the acid was added at the top corner, the mesoporosity was completely lost and the samples became entirely microporous. These results clearly show that mixing is playing a key role in product properties via influencing the particle formation pathways. Interestingly, as both microporous and mesoporous silicas are desired in applications, it seems possible to control pore size distribution via mixing conditions.

**Figure 4:**
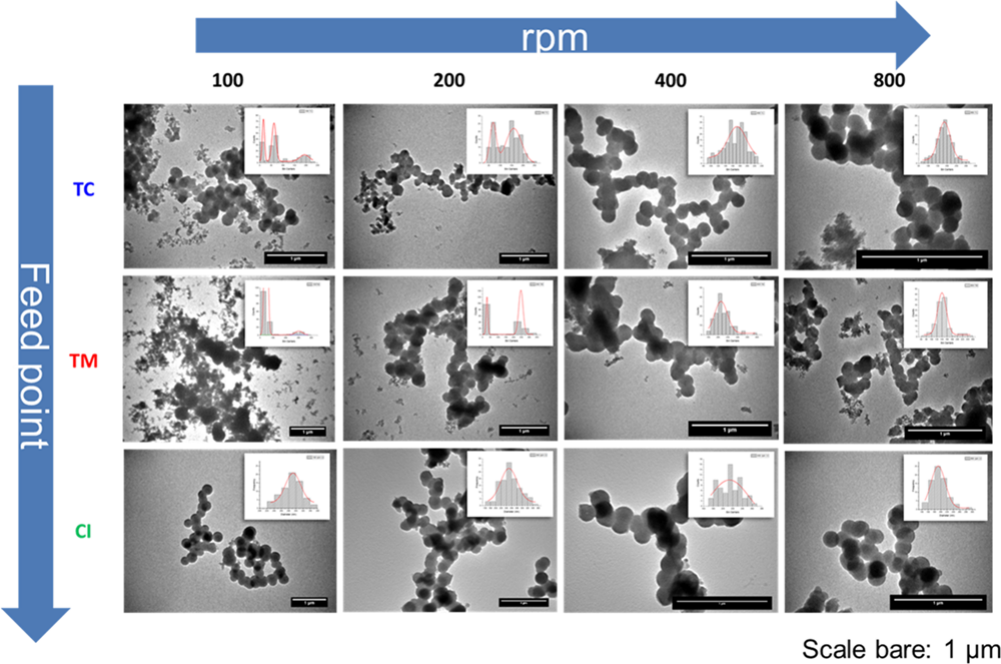
TEM micrographs and particle size distribution plots (insets) for BIS produced under varying conditions of impeller speed and feed location (also see Figure S2 for larger particle size distribution plots).

Using transmission electron microscopy, we also measured the secondary particle sizes and size distributions (Figures 3d, 4, and S2). While for the top-corner feed location, the particle sizes did not change as a function of impeller speed (gray dashed line in Figure 3d), they changed for the other feeding points (TM and CI)—the particle sizes were found to decrease with increasing stirring speeds (blue arrows in Figure 3d). For TC addition, it seems that the oligomer conversion to particles and particle breakage is less sensitive to changes in local turbulent fluctuation (feeding point), with particle formation driven mainly by diffusion, thus leading to unchanged particle size. For the TM and CI locations, it is likely that under these higher turbulent conditions, there is a reduced rate of oligomer conversion to particles and higher breakage.^70^ When considering the effects of stirring (or turbulence), as expected, under low turbulence, the particle size distribution (insets in Figure 4) was broad or multimodal (e.g., for 100 rpm). With increasing the turbulence, particle size distribution became narrower as well as the particles were found to be well-defined sphere.

In summary, following the workflow shown in Figure 1, we found that both the stirring speed and feed location affected the oligomer–particle interconversions, which also impacted the pore sizes, particle sizes, and size distributions. These results strongly suggest that the timescales of BIS synthesis and mixing are comparable, and these results have identified that there are likely interactions between these two processes (discussed further in the Quantitative Mixing Analysis section). It is therefore clear that BIS synthesis is controlled by mesomixing and, to a lesser extent, micromixing. While micromixing controlled systems are well known, BIS presents a unique scenario, where established methodologies are unlikely to provide accurate descriptions of the BIS synthesis as it follows nonclassical formation pathways and traditional methods focus on particle growth and ignore the earlier stages (i.e., the nonclassical features), such as pre-nucleation clustering, cooperative assembly between organic additives and inorganic species, and oligomerization reactions.^71,72^

Specifically, for reactive heterogeneous systems, there is a gap in the literature on modeling and predicting reactive mixing for reactions involving multistep synthesis (i.e., nonidealized reactions that are not spontaneous and their timescales are comparable to mixing timescales). Indeed, recent work by Villermaux and co-workers has acknowledged that it is still challenging to understand the impact of mixing on multistep reactions, and hence, they have resorted to the use of idealistic homogeneous reactions.^73^ Similarly, given the challenges in handling complex reactive systems, Wojtas and co-workers also limited their reactive mixing experiments to simplistic homogeneous neutralization reactions with well-known kinetics.^74^ Inherently, there is still a long way to adopt such methods to real reactions, such as BIS synthesis. This further highlights the importance of the need for a novel methodology to build empirical scale-up models.

As such, to study the effects of mixing on nanomaterial synthesis, we developed an in-house method for image acquisition and analysis to calculate the global and local mixing times and overall degree of mixing (DoM). This new technique was then applied to study the mixing kinetics for bioinspired silica (BIS) formation reactions over a wide range of mixing scenarios. The results were used to formulate the correlation between the mixing time and fluidic conditions. Below, each of these aspects is presented in separate sections.

### Method Development and Validation

**Figure 5:**
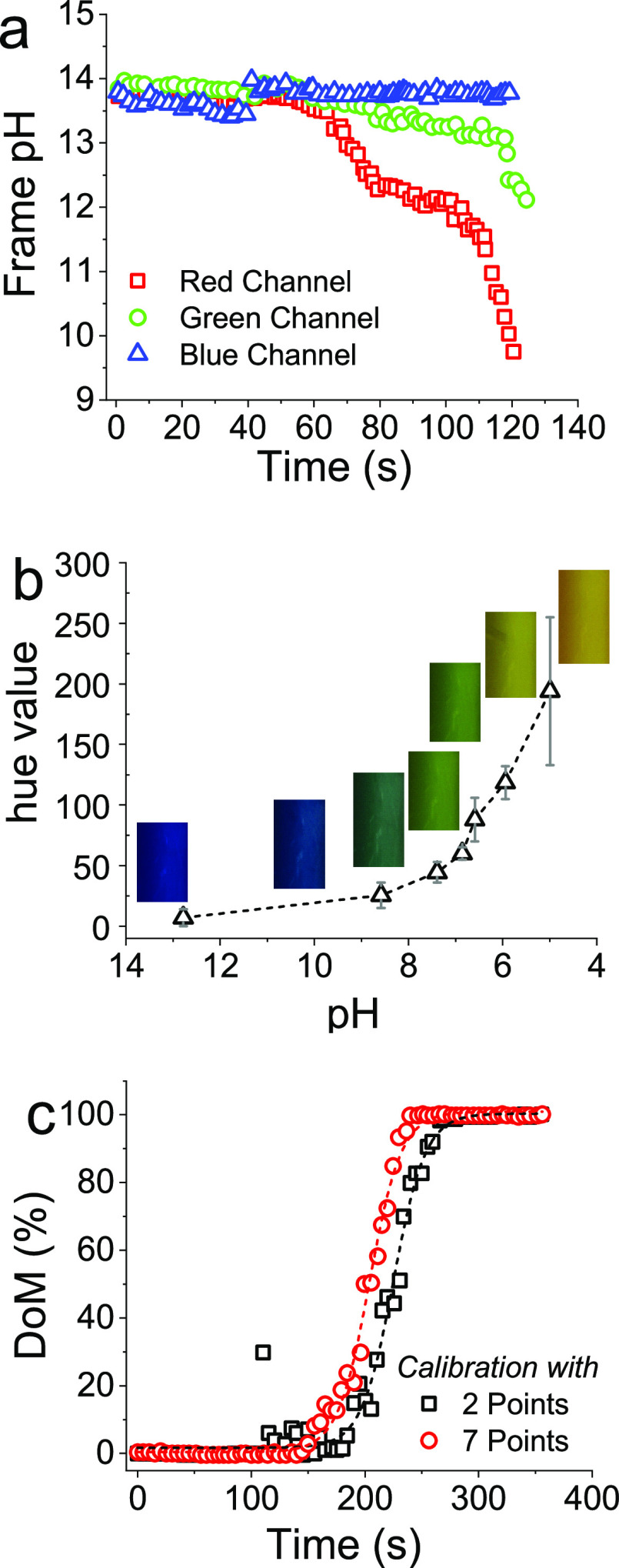
(a) Effect of pH on the sensitivity of RGB channels. (b) Color/hue value calibration with the pH. (c) Effect on DoM estimation as a function of calibrations.

An initial evaluation was performed to determine which of the RGB color channels was best suited in assessing the color change during the reaction. Figure 5a shows the evolution of the three RGB channels as the indicator color in the vessel changes from blue to green in a typical experimental condition. As can be seen, the blue channel is fairly constant, showing that it is rather insensitive to a change in pH; consequently, it is unsuitable for use in monitoring reaction pH. On the other hand, the red and green channels show appreciable sensitivity, with red being the most sensitive. This is consistent with the literature, where the red channel was reported to be more receptive to indicate the color change in a mixing vessel;^55^ hence, the red channel was selected for further analysis.

The pH of the reaction mixture changes from an initial value of 13 (blue) down to 7 (green) upon the addition of the acid and homogenization. To convert this color change into pH values within each frame, a two-point calibration between pH 13 and pH7 was used to estimate corresponding DoM as a function of time. However, until the acid is mixed, localized variations in the acid concentration lead to certain zones in the reactor reaching a pH as low as 5. Further, the change in color (or pH) was observed to be gradual and hence a 2-point calibration may not be adequate. Therefore, a 7-point calibration was constructed (Figure 5b), and the peak location of the histogram or the midpoint of the histogram was used to obtain a corresponding pH value (see Table S1).

The evolution of DoM over time from both calibrations was calculated as shown in Figure 5c. It can be observed that there was a degree of randomness associated when the 2-point calibration was used while little to no such variations were observed for the 7-point calibration. Additionally, when using the 2-point calibration, a time lag was observed to reach the given DoM when compared with the 7-point calibration. Therefore, the 7-point pH color calibration was preferred to map out the pH values and further DoM analyses.

We then investigated the effect of grid sizes within an RoI on the degree of mixing. While a higher resolution grid resulting in a large number of grids is ideal from accuracy standpoint, it can make the calculations onerous. Making the grid coarser and hence reducing the total number of grids can cause large errors. Grid sizes of 100 × 100, 50 × 50, 30 × 30, 20 × 20, 10 × 10, and 5 × 5 were considered, and the corresponding DoM was plotted as a function of mixing time (see Figure S3 and Table S3). Analysis of the mixing revealed that the degree of scatter in the data was more pronounced as grid sizes increased (e.g., for grid sizes of 5 × 5 and 10 × 10). Subsequently, the *t*_m_ values calculated varied significantly from a grid size of 5 × 5 to 30 × 30. However, it was observed that changes to *t*_m_ for 30 × 30 grid size and beyond was relatively insignificant; hence, 30 × 30 grid size was chosen.

**Figure 6:**
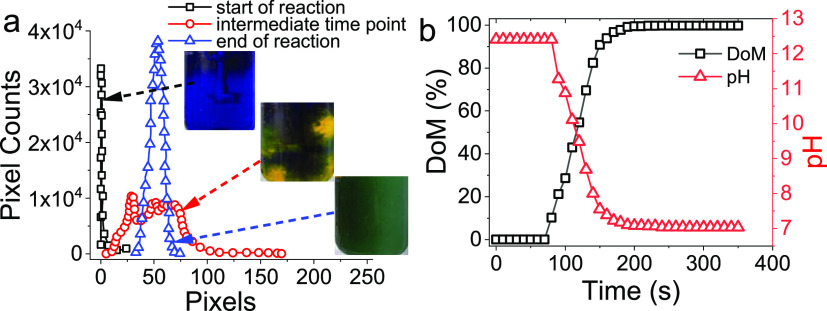
(a) Representation of the reaction mixture at three different time points during the reaction and corresponding histogram plots with a notable color change from blue to green. (b) DoM and pH evolution as a function of time.

Next, preliminary BIS synthesis experiments were carried out to ensure the reliability of the methodology. Figure 6a shows representative frames obtained from a video recording at different times as the reaction progresses and corresponding histograms from the red channel. This information was used to calculate the dynamic behavior of DoM and pH (Figure 6b), which exhibits an S-shape sigmoid from 0% DoM to 100% with pH decreasing from 13 to 7 as the stirring effect sets in. Although reported for a different system, a similar profile for the mixing curve was previously reported.^56,61,75^

### Quantitative Analysis of Mixing

**Figure 7:**
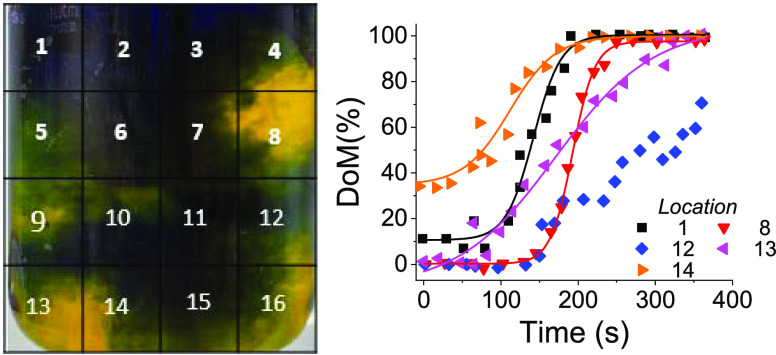
Profiles of local mixing for selected locations (shown in left).

Colorimetric methods using high-resolution image analysis are powerful to study local and global mixing. It is well known that for stirred tanks, there is a spatial variation in the degree of mixing, at least initially, as noted by many researchers.^55,56^ As such, the technique developed herein was initially tested to identify local mixing effects. The reactor images were divided into 4 × 4 mesh (see Figure 7) and for each of the resulting 16 locations, the evolution of DoM was monitored (Figure 7). It can be seen that homogeneity across the vessel was unevenly distributed with each location, exhibiting the difference in the lag time, mixing rates, and the final DoM. These observations highlight the capability of our technique to monitor and quantify localized mixing, and future work is focused on understanding the spatial and temporal mixing in BIS synthesis.

**Figure 8:**
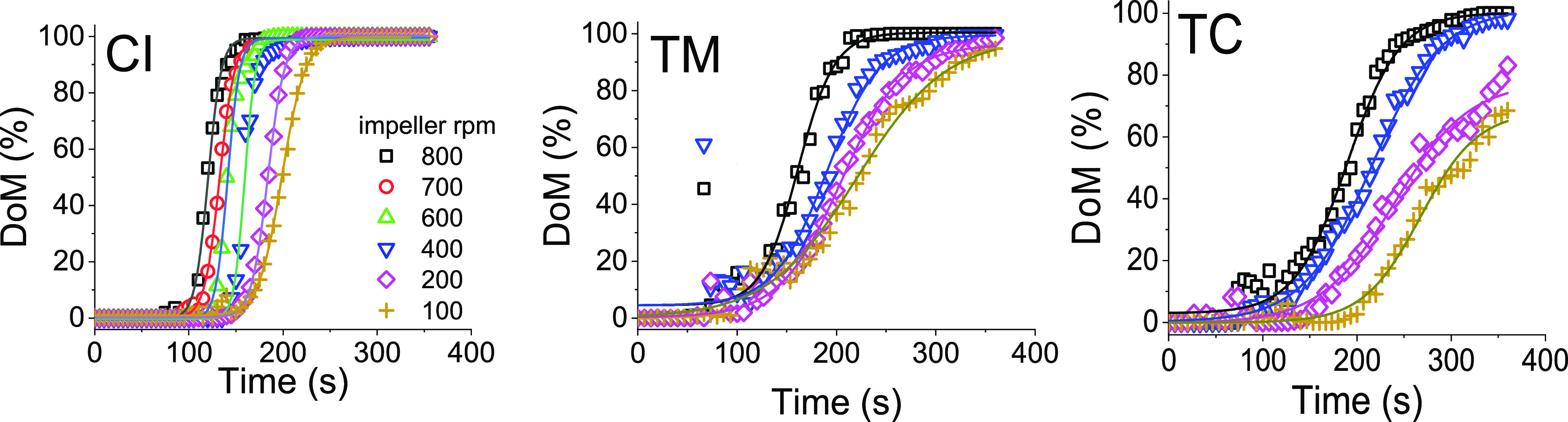
Dynamic mixing profiles obtained upon varying the acid feed location as close-to-impeller (CI), top-middle (TM), and top-corner (TC). Solid lines show a sigmoidal fit.

Next, we used the image analysis method for measuring the global mixing time for the entire reactor. By recoding the videos of the reactor over the duration of the reaction and using the image analysis described above, we calculated the DoM as a function of reaction time by varying the location of feed (acid) addition and the impeller rotational speed. The feed locations selected were close-to-impeller (CI), top-middle (TM), and top-corner (TC) as shown in Figure 1. These locations represent zones in the reactor with varying degrees of mixing, going from the best mixing at CI to the worst at TC. The impeller rotational speed was varied between 100–800 rpm (*Re* ≈ 4000–33,000). Figure 8 shows the dynamic DoM for varying impeller rotational speed and three feed locations. As established above, the DoM plots depicted an S-shaped sigmoidal curve, which is also consistent with the literature for single-impeller cylindrical stirred tanks.^20,21,53^ These DoM data were fitted with the Boltzmann sigmoidal model as shown by solid lines in Figure 8 (the fit parameters for all conditions explored are given in Table S2). From this data, it is clear that, as expected, the impeller speed and feed locations have significant impacts on DoM. While for the cases where feed was added close-to-impeller (CI) or top-middle (TM), complete mixing was always achieved (DoM reaching 100%), when feed was added at top-corner (TC), poor mixing was evident with DoM not reaching 100% for lower impeller speeds.

**Figure 9:**
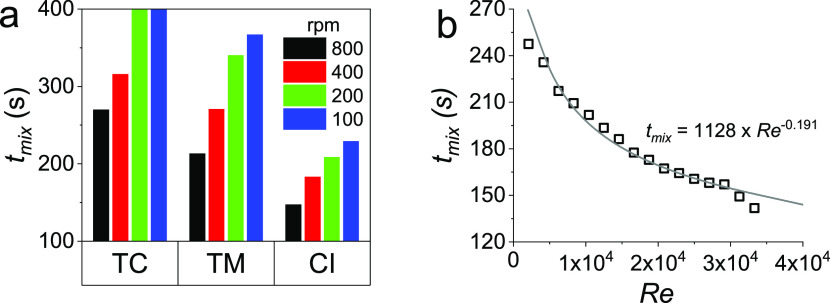
(a) Experimental mixing times obtained from different feed locations (top-corner (TC), top-middle (TM) and close-to-impeller (CI)) and impeller speeds (100–800 rpm). (b) Experimental mixing time as a function of *Re* (open symbols), the solid line shows a power model fit (*R*^2^ = 0.95). Note for TC 100 and 200 rpm, the system did not reach 100% DoM, hence *t*_m_ → ∞.

To quantify these behaviors, next, using the fit parameters, we calculated the mixing times (time to reach 95% DoM) for each of these situations (see Figure 9a). As expected, the close-to-impeller (CI) condition required shorter time to achieve complete mixing (typically 150–240 s) compared to the other feed locations (between 220–360 s). The top-corner condition exhibited the worst mixing scenario, with incomplete mixing (DoM of 70 and 60%) observed for 200 and 100 rpm rotational speeds, respectively. For a given feed location, again as expected, the increase in impeller rotational speed reduced the mixing time. Nonreactive mixing times measured for our reactor range around tens of seconds, which is comparable to literature data (modeled or experimentally measured) for similar tank sizes and geometries for a range of impellers/stirrers.^33,38,56,76^ This mixing time is about 10-fold shorter than those measured herein even when accounting for the fact that we have used an unbaffled stirred tank. We note that, however, these literature data (e.g., that presented in Table 5 of Nere et al.^33^) were limited to homogeneous liquid phase systems with no reactions or precipitation involved, while for a reactive system, *t*_mix_ of several minutes were reported for unbaffled stirred tanks (e.g., see ref (68)). These observations further support the strong interactions between the mixing and BIS synthesis processes identified above and confirm mesomixing as the dominant mixing mechanism. Decoupling mixing and synthesis when modeling the process will introduce significant errors due to not including the interplay. Therefore, in the absence of detailed kinetics models, to enable BIS scale-up, using the “lumped” mixing times appears to be more valuable. As such, we used this overall mixing time for generating scale-independent rules for the BIS system, which can help design larger reactors that can offer the right mixing scenarios for the desired materials synthesis/properties.

Given the mixing theory discussed in the Introduction section, and especially the relation between the mixing time and *Re*, we sought to explore this correlation for our results. Figure 9b shows that the mixing time decreases with *Re*, consistent with the correlation developed by Norwood and Metzner and other literature studies.^34,39^ Using eq 6, for our system, where the geometries of the impeller and the reactor were fixed, a correlation of *t*_mix_ with Reynolds number can be described with a power law as given by eq 12, where *A* is the geometry-dependent parameter.
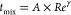
12

Figure 9b shows a good fit to eq 12 (*R*^2^ = 0.95), yielding the exponent as −0.191 with standard deviation between the experimental mixing time and those predicted by the correlation are in the range of 10% (not shown). Previous research on correlating mixing time with *Re* has shown similar observations with γ varying between −0.1 and −0.75.^38,39,77^ This wide range is attributed to the variations in impeller designs, reactor types, *Re* investigated, and the type of measurements used for the mixing time.^78^ This robust and scale-independent correlation can form the basis for upscaling the synthesis of BIS by maintaining the mixing time to obtain consistent product quality. This can be achieved using suitable reactor and impeller designs for a given scale.

## Conclusions

In this experimental investigation, a new hybrid pH-colorimetric method, which relies on image analysis and pH measurement, has been developed to characterize meso- and macromixing of stirred vessels. This method only requires a high-speed digital camera, a pH probe, and image analysis for the characterization of the degree of mixing. This method circumvents the subjectivity of the colorimetric method for mixing time determination, which is subject to investigator’s interpretation. Here, we presented the validation of this method and demonstrated its robustness in applying for bioinspired silica synthesis. We were able to obtain information on the spatial and temporal distribution of the mixing, thereby enabling the identification and quantification of mixing mechanisms. A new correlation between the mixing time and Reynolds number was developed herein for bioinspired silica synthesis, which can be used to design sustainable manufacturing of nanomaterials—this is first of a kind for green nanomaterials. We believe that the methodology developed herein is applicable for wider nanomaterial synthesis. Future research is focusing on a detailed understanding of the spatial and temporal mixing, the measurements of the kinetics, and the use of computational modeling to develop scale-up rule.

## Supporting Information Available

pH—color map; fit parameters from the parametric study; silicate speciation under different conditions; particle size distributions; and effect of grid size (PDF)
