# Supplementary material for: A Novel Method for Understanding the Mixing Mechanisms to Enable Sustainable Manufacturing of Bioinspired Silica
Source: ACS Eng Au. 2022 Nov 16;3(1):17–27. doi: 10.1021/acsengineeringau.2c00028 (PMC9936550; doi:10.1021/acsengineeringau.2c00028)
Supplement: Supplementary file 1 — eg2c00028_si_001.pdf [file eg2c00028_si_001.pdf]

Supporting Information

A novel method for understanding the mixing mechanisms to enable sustainable manufacturing of bioinspired silica

*Yahaya D. Baba, Mauro Chiacchia and Siddharth V. Patwardhan**
Green Nanomaterials Research Group,
Department of Chemical and Biological Engineering,
University of Sheffield, Mappin Street, Sheffield S1 3JD, U. K.
* s.patwardhan@sheffield.ac.uk

Table S1: Summary of pH value mapped against colour change from blue to yellow used for calibration.

Colour spectrum pixel/ hue values	Histogram plots and corresponding image	Measured pH values
0-14	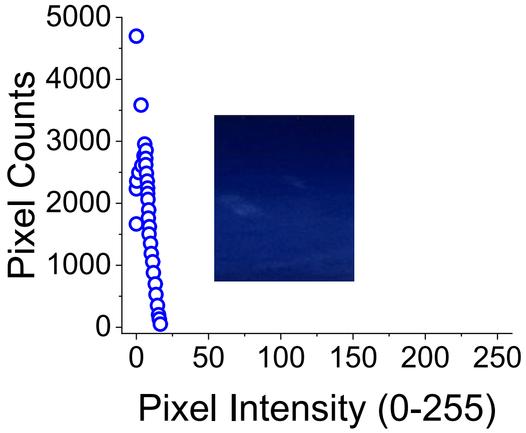	12.78
15-36	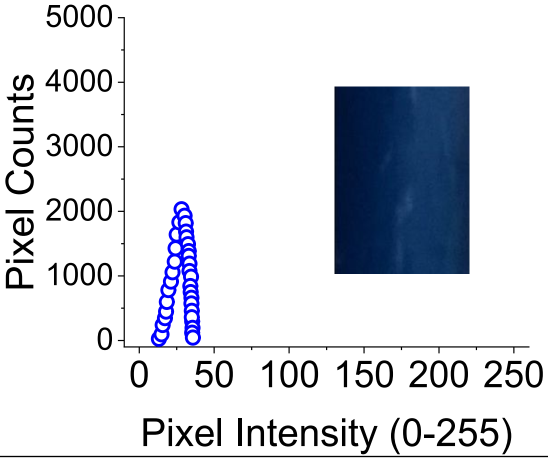	8.58
36-53	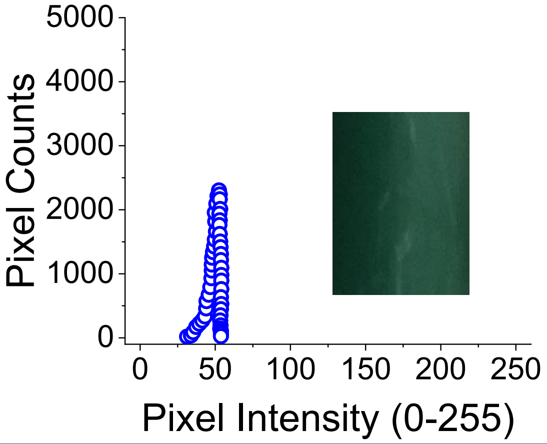	7.39

55-67	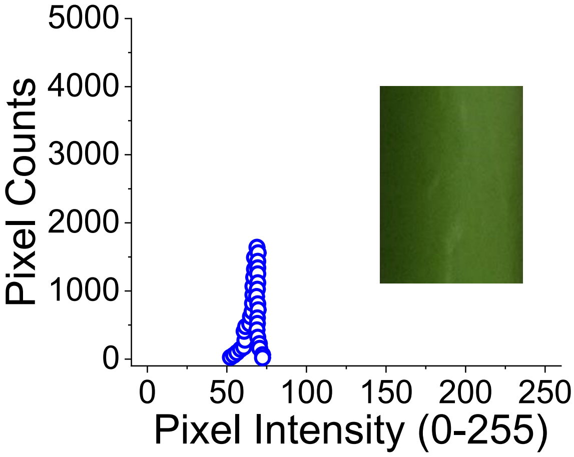	6.855
70-106	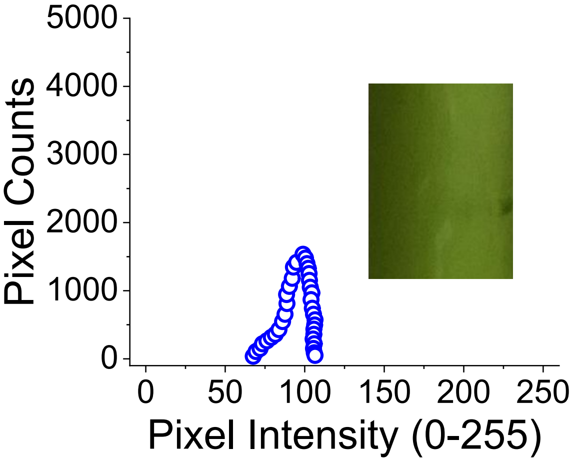	6.59
105-132	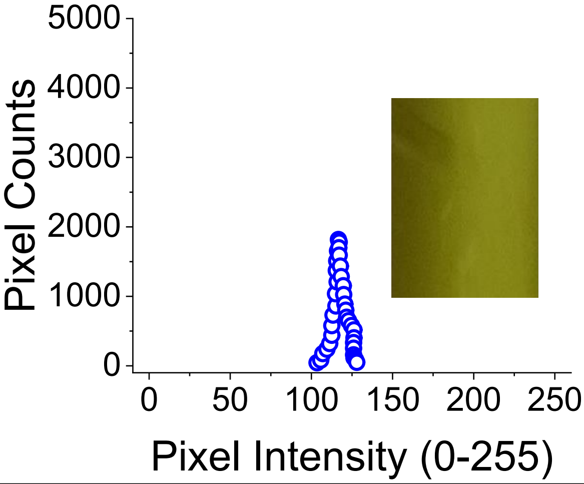	5.94
>133	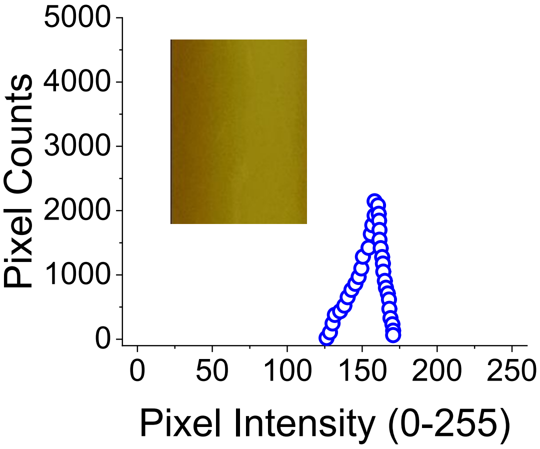	4.99

Table S2. Fit parameters from the parametric study for each feed location and varying stirring speeds.

**Feed location: Close to Impeller (CI)**					
rpm	t_o	A_1	A_2	Δx	t_{mix} *(sec)*
800	120.44	-0.277	99.44	6.99	141.9
750	128.14	0.38	99.91	7.13	149.4
700	132.713	0.08	99.41	7.95	157.1
650	140.01	-0.15	100.11	7.15	158.1
600	141.22	-0.43	99.433	6.34	160.7
550	142.9	0.11	100.01	6.9	164.3
500	147.98	0.19	100	6.4	167.3
450	153.11	-0.18	99.99	6.5	173.0
400	159.31	-0.23	98.823	5.68	177.6
350	165.14	0.012	98.99	6.7	186.3
300	171.55	-0.88	100	7.3	193.5
250	177.55	0.11	100.19	8.13	201.8
200	183.96	0.16	100.26	8.79	209.4
150	193.85	0.01	99.98	8.1	217.2
100	198.86	0.91	99.68	12.304	235.8
50	218.23	0.15	99.78	9.9	247.6
**Feed location: Top Middle (TM)**					
rpm	t_o	A_1	A_2	Δx	t_{mix} *(sec)*
800	161.36	4.55	100.39	18.39	213.22
400	190.88	4.48	99.51	26.57	270.53
200	204.99	0.34	96.21	31.00	340.13
100	220.86	0.13	98.73	45.11	366.84
**Feed location: Top Corner (TC)**					
rpm	t_o	A_1	A_2	Δx	t_{mix} *(sec)*
800	189.65	2.96	100.15	27.85	269.94
400	215.93	0.22	101.29	36.80	315.75
200	240.00	-0.34	77.83	39.88	--
100	267.35	-0.03	68.22	30.47	--

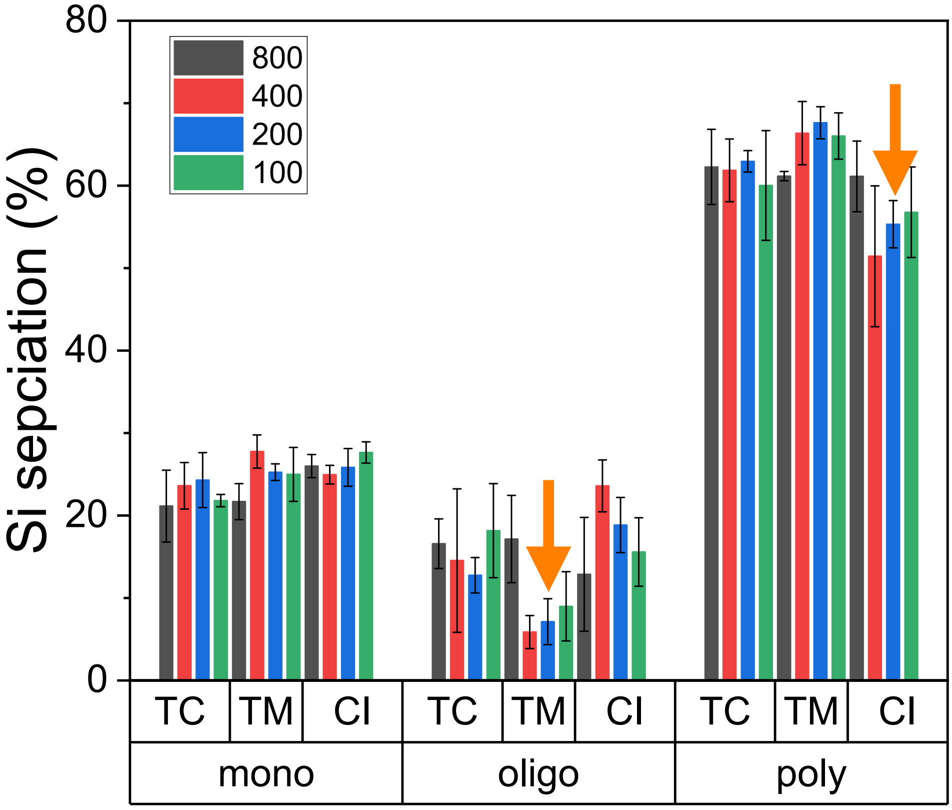

Figure S1. Silicate speciation (monomers, oligomers and polymers) as a function of stirring speed and the location of feed addition. Orange arrows show key changes in the speciation.

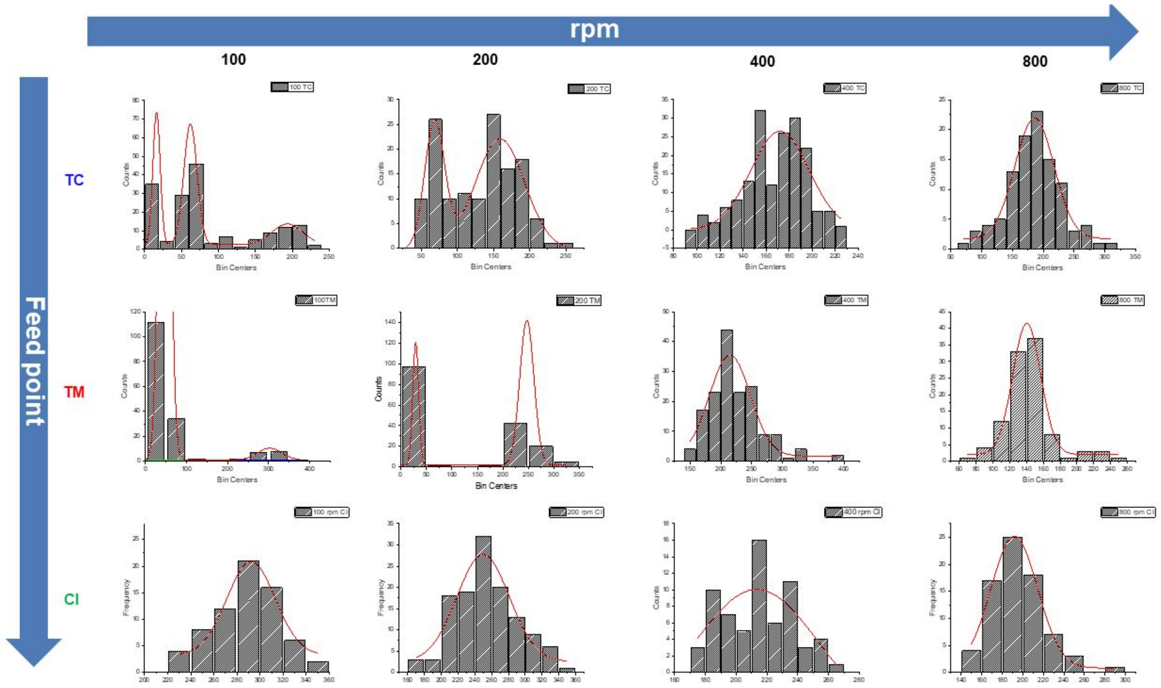

Figure S2: Particle size distributions for the TEM data for different fluidic conditions.

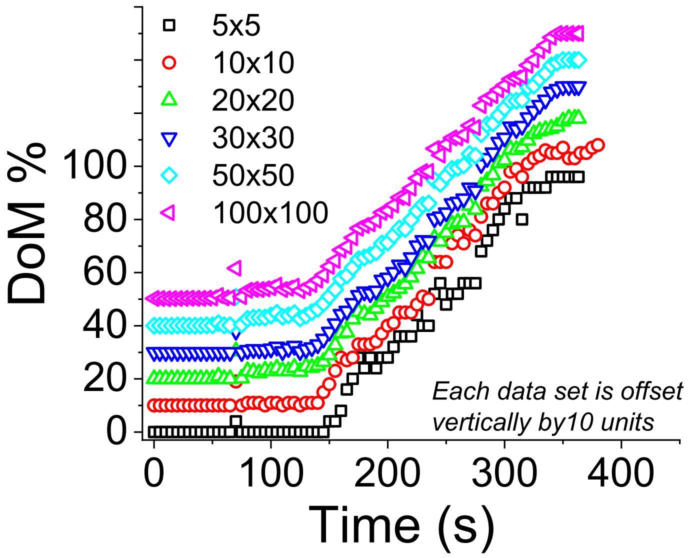

Figure S3: A study to compare the effects of Region of Interest RoI grid size on the Degree of Mixing (DoM) computation. Data sets are vertically offset by 10 units from the previous set in order to avoid overlap.

Table S3. Fitting parameters to compare the effects of grid size on DoM.

**Grid Sizes**	R^2	t_o	A_1	A_2	Δx	t_{mix} *(sec)*
5x5	0.98	250.34	-3.27	101.39	49.56	384.94
10x10	0.99	240.76	-1.87	102.55	43.48	359.96
20x20	0.99	246.04	-1.89	108.94	49.41	341.83
30x30	0.99	254.78	-2.57	113.46	49.55	337.27
50x50	0.99	248.72	-2.51	113.39	52.91	336.98
100x100	0.99	245.83	-2.69	112.98	54.33	337.78
